# Dammarane-type Triterpene Saponins from the Flowers of *Panax notoginseng*

**DOI:** 10.3390/molecules14062087

**Published:** 2009-06-10

**Authors:** Jing-Rong Wang, Yuko Yamasaki, Takashi Tanaka, Isao Kouno, Zhi-Hong Jiang

**Affiliations:** 1 School of Chinese Medicine, Hong Kong Baptist University, Kowloon Tong, Hong Kong, China; 2 Department of Natural Product Chemistry, Graduate School of Biomedical Sciences, Nagasaki University, Bunkyo-machi 1-14, Nagasaki 852-8521, Japan

**Keywords:** *Panax notoginseng*, dammarane-type, saponins, ginsenoside

## Abstract

Four new dammarane-type triterpene saponins named floranotoginsenosides A (**1**), B (**2**), C (**3**) and D (**4**), together with five known triterpene saponins, were isolated from the flowers of *Panax notoginseng*. Their structures were elucidated on the basis of spectral and chemical evidence.

## 1. Introduction

Root of *Panax notoginseng* (Burk) F. H. Chen (Araliaceae), commonly referred to notoginseng and “*San-Qi”*, is a well-known medicinal herb, used historically in traditional Chinese medicine as a hemostatic agent that both invigorates and builds blood. The flowers of *P. notoginseng* have been used for treating hypertension, vertigo, tinnitus and laryngopharyngitis [1]. The saponin fraction of the notoginseng flowers has been proven to have hepatoprotective effect [2]. More than 60 dammarane-type saponins named ginsenosides, notoginsenosides and gypenosides have been hitherto isolated from this plant [3,4], among which about 20 saponins were obtained from its flower buds [5,6]. 

We herein report the isolation and structural elucidation of four new dammarane-type triterpene saponins **1-4**, together with five known saponins **5-9** from the flowers of *Panax notoginseng.*

## 2. Results and Discussion

The water layer of methanolic extracts of notoginseng flowers was separated by MCI gel CHP 20P and ODS column chromatographies, and purified by preparative HPLC (ODS) to afford compounds **1**-**9**. Compounds **5-9** were identified as gypenoside LXIX (**5**) [7], 3*β*,12,20*S*-trihydroxy-25-hydro-peroxydammar-23-ene-3-*O*-[*β*-D–glucopyranosyl(1→2)-*β*-D-glucopyranosyl]-20-*O*-[*β*-D-xylopyrano-syl(1→6)]-*β*-D-glucopyranoside (**6**) [8], notoginsenoside FP_2 _(**7**) [4], floraginsenoside O (**8**) [9] and gypenoside LXXI (**9**) [10], respectively, by comparison of their spectral data with those described in the literature. Compounds **7** and **8** were previously isolated from *Panax notoginseng* and *P. ginseng*, respectively, while compounds **5**, **6** and **9** were isolated from *Gynostemma pentaphyllum.*

**Figure 1:**
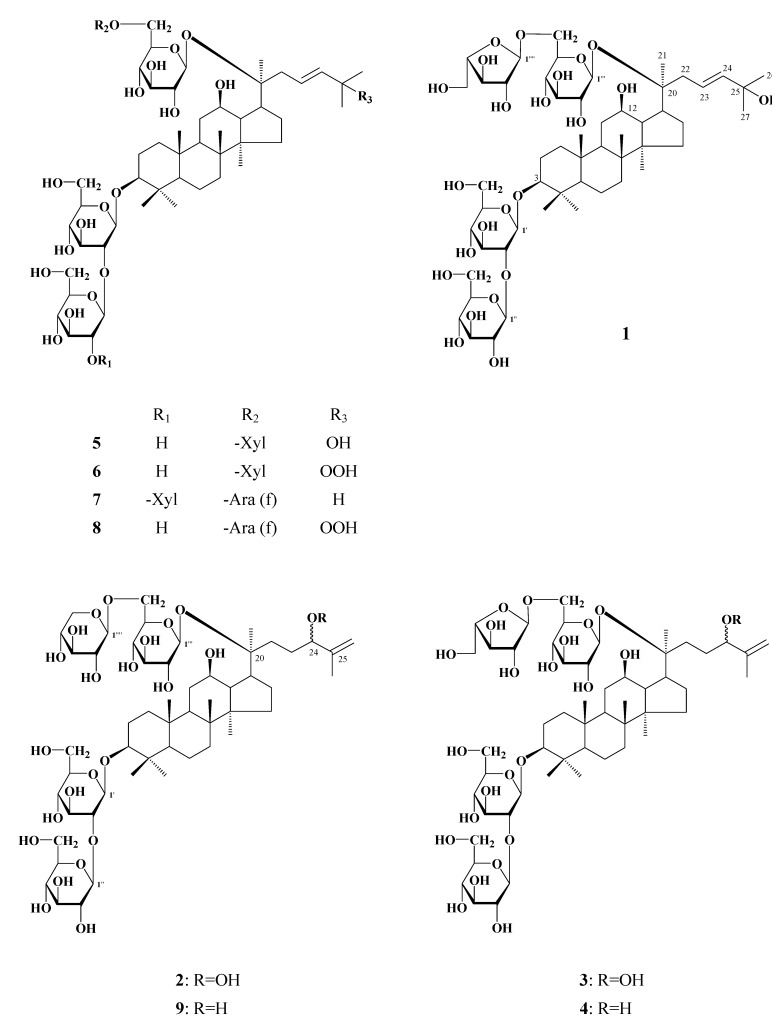
Structures of compounds **1-9.**

Compound **1**, named floranotoginsenoside A, was obtained as a white powder, [α]^D^_25_: -4.93^o ^(MeOH). The molecular formula of **1** was determined to be C_53_H_90_O_23_ on the basis of its negative FAB-MS (*m/z* 1,093 for [M-H]^-^) and high-resolution ESI-MS data (*m/z* 1,093.5790 for [M-H]^-^). The^ 1^H-NMR spectrum of **1** showed signals assignable to eight singlet methyls (*δ*_H_ 0.86, 0.91, 0.93, 1.02, 1.07, 1.29 × 2, 1.33) together with four anomeric proton signals at *δ*_Η_ 4.94 (s), 4.82 (d, *J* = 8 Hz), 4.66 (d, *J* = 8 Hz), 4.60 (d, *J* = 8 Hz). In the ^13^C-NMR spectrum of **1**, 30 carbon signals due to the aglycone moiety were in good agreement with those of **5, **especially the olefinic carbon signals (*δ*_C_ 123.8 and 141.9), and the oxygenated carbon signal (*δ*_C_ 71.5) of the side chain, suggesting that the aglycone of compound **1** is the same as that of **5**, in which a double bond located at C-23, 24 and a hydroxyl group attached to C-25. The signals at *δ*_C_ 91.3 and 84.6 indicated that **1** is a bisdesmoside. Besides the aglycone’s ^13^C-NMR signals, the remaining carbon signals of compound **1** were found to be superimposable on those of compound **5** except for the presence of signals derived from an *α*-L-arabinofuranosyl moiety instead of *β−*D-xylopyranosyl moiety in **5**, suggesting that oligosaccharide chain of *α*-L-arabinofuranosyl-(1→6)-*β*-D-glucopyranosyl attached to 20-OH of the aglycone. This was further supported by chemical shift comparison of these sugar signals with those of compound **8**. On the basis of these results, the structure of floranotoginsenoside A was assigned to formula **1 **(Figure 1). 

Floranotoginsenoside B **(2)** was also obtained as a white powder, whose negative FAB-MS showed a quasimolecular ion peak at *m/z* 1,091 ([M-H-H_2_O]^-^). The molecular formula C_53_H_90_O_24_ was determined by high-resolution ESI-MS (*m/z* 1,109.5748 for [M-H]^-^). The ^1^H-NMR spectra of 2 showed four anomeric proton signals at *δ*_Η_ 5.38 (d, *J* = 8 Hz), 5.05 (d, *J* = 8 Hz,), 5.00 (d, *J* = 8 Hz), 4.92 (d, *J* = 8 Hz), a vinyl methyl [*δ*_Η_ 1.96 (s, H_3_-27)], and a methine proton bearing a hydroperoxyl group [*δ*_Η_ 4.79 (dd-like, H-24)] which was supported by chemical shift comparison with those of related hydroperoxylated triterpenes [11]. The ^1^H-NMR and ^13^C-NMR of **2** are quite similar to those of notoginsenoside-C [12], except for the presence of signals for a *β*-D-xylopyranosyl moiety and absence of signals for a* β*-D-glucopyranosyl moiety. This suggested that **2** has the same aglycone and similar glycosylation manner with that of notoginsenoside-C with the only difference in the *β*-D-xylopyranosyl moiety as the terminal sugar of C-20 in **2**. Reduction of **2** by triphenylphosphine afforded gypenoside LXXI (**9**), which strongly supports the proposed structure. Thus the structure of floranotoginsenoside B was characterized as shown for **2** (Figure 1). 

Floranotoginsenoside C (**3**) was isolated as a white powder and has the same molecular formula as 2 on the basis of the [M-H-H_2_O]^-^ peak at *m/z* 1,091 in the negative FAB-MS and [M-H]^-^ at *m/z* 1,109.5747 in negative HR-ESI-MS. Its ^1^H-NMR and ^13^C-NMR spectral data again suggested a side chain with a hydroperoxyl group at C-24, as evidenced by the signals ascribed to the vinyl methyl [*δ*_Η_ 1.97 (s, H_3_-27)], and methine group bearing hydroperoxyl group [*δ*_Η_ 4.79 (dd-like, H-24), *δ*_C_ 90.1 (C-24)]. Comparison of the ^1^H-NMR and ^13^C-NMR spectra of **3** with those of **2** revealed that the only difference was the replacement of the terminal *β*-D-xylopyranosyl group in C-20 position in **2** by an *α*-L-arabinofuranosyl moiety in **3**. Based on these evidences, structure **3** was assigned to floranoto-ginsenoside C (Figure 1). 

Floranotoginsenoside D (**4**) has the molecular formula C_53_H_90_O_23_ which is one oxygen less than **3**, on the basis of the [M-H]^- ^peak at *m/z* 1,093 in the negative FAB-MS and *m/z* 1,093.5792 for [M-H]^- ^in negative HR-ESI-MS spectra. It’s ^1^H-NMR and ^13^C-NMR spectra closely resembled those of **3**, especially the signals arising from the sugar moiety and the rings A-D, indicating that **4** is a also dammaranes bidemoside. In comparison of the ^13^C-NMR spectra of **4** with that of **3**, the upfield shift of C-24 (Δ*δ*_C_ -13.7) and C-26 (Δ*δ*_C_ -2.9), as well as the downfield shift of C-25 (Δ*δ*_C_ +3.4) were observed, indicating that a hydroxyl group is located at C-24 position in **4**. This was supported by chemical shift comparison of H-24 (*δ*_H_ 4.42) and C-24 (*δ*_C _76.4) with that of **9** and related compounds with the same side chain structure [13]. When 3 was treated by triphenylphosphine, **4** was obtained. Therefore, the structure of floranotoginsenoside D was determined to be the formula **4** (Figure 1). 

**Table 1:** ^13^C-NMR data for compounds **1-9**.

	1^a)^	2^c)^	3^ c)^	4^ c)^	5^a)^	8^a)^	9^ c)^
C-1	39.9	39.2	39.2	39.2	40.2	40.2	39.2
C-2	27.0	26.8	26.7	26.7	27.0	27.0	26.7
C-3	91.3	89.0	89.0	89.0	91.3	91.3	89.0
C-4	39.8	40.0	39.7	39.7	40.5	40.6	40.0
C-5	57.6	56.4	56.3	56.3	57.5	57.5	56.4
C-6	19.2	18.4	18.4	18.4	19.2	19.2	18.6
C-7	35.8	35.1	35.1	35.0	35.8	35.8	35.1
C-8	40.2	40.0	40.0	40.0	41.0	40.6	40.0
C-9	50.0	50.2	50.2	50.1	51.0	52.5	50.2
C-10	37.9	36.9	36.9	36.9	37.9	37.9	36.9
C-11	30.9	30.9	30.9	30.9	30.9	30.9	30.9
C-12	71.3	71.1	70.3	70.4	71.5	72.0	71.1
C-13	50.0	49.5	49.4	49.3	49.8	52.5	49.4
C-14	51.0	51.4	51.4	51.4	52.4	51.0	51.4
C-15	30.1	30.7	30.7	30.8	31.3	31.3	30.8
C-16	27.2	26.6	26.6	26.7	27.2	27.2	26.7
C-17	52.5	51.5	51.5	52.0	53.1	53.1	51.8
C-18	16.4	16.0	16.0	15.8	16.7	16.4	16.0
C-19	16.7	16.3	16.3	16.3	16.7	16.7	16.3
C-20	84.6	83.4	83.4	83.5	84.6	84.6	83.4
C-21	23.4	22.4	22.4	22.5	23.3	23.4	22.6
C-22	39.9	32.7	32.7	32.7	40.0	41.0	32.7
C-23	123.8	26.3	26.5	30.4	123.8	127.5	30.7
C-24	141.9	90.1	90.1	76.4	141.9	138.1	76.7
C-25	71.5	146.2	146.1	149.5	71.5	82.6	149.4
C-26	31.4	113.3	113.4	110.5	30.1	24.8	110.0
C-27	31.4	17.4	17.7	18.0	29.8	25.2	18.4
C-28	28.4	28.1	28.1	28.1	28.4	28.4	28.1
C-29	16.7	16.6	16.6	16.6	16.7	16.7	16.6
C-30	17.3	17.4	17.3	17.2	17.3	17.3	17.3
Glc-1'	105.4	105.1	105.1	105.1	105.3	105.4	105.1
2'	81.1	83.4	83.3	83.3	81.0	83.2	83.7
3'	78.5	78.3	78.3	78.2	78.4	78.3	78.2
4'	71.9	71.6	71.6	71.6	71.8	71.5	71.6
5'	78.1	78.1	78.3	78.2	78.0	78.1	78.1
6'	62.8	62.9	62.9	62.8	63.0	62.8	62.8
Glc-1''	104.5	106.0	106.0	106.0	104.4	105.4	106.0
2''	77.7	77.1	77.9	77.9	77.6	77.6	77.1
3''	77.9	78.1	78.1	78.9	77.8	77.8	78.1
4''	71.9	71.6	72.0	72.0	71.6	71.9	71.7
5''	78.3	78.3	78.8	78.9	78.2	78.1	78.2
6''	63.1	62.7	62.7	62.7	62.8	63.0	62.6
Glc (Xyl)-1'''	98.1	98.0	98.0	98.0	98.0	98.0	98.0
2'''	75.3	75.0	74.9	75.0	75.3	75.3	75.9
3'''	78.5	79.3	79.2	79.0	78.4	78.9	79.2
4'''	71.5	71.6	71.6	71.6	71.1	71.5	71.6
5'''	77.7	77.1	77.1	77.1	77.5	77.6	77.1
6'''	68.5	70.0	68.3	68.3	70.1	63.0	69.8
Ara (Xyl, Glc)-1''''	110.0	105.6	110.0	110.0	104.4	110.0	105.6
2''''	83.3	74.8	83.4	83.5	74.8	83.2	74.8
3''''	78.5	78.3	78.8	78.9	78.4	78.5	78.2
4''''	85.7	71.1	85.9	85.9	71.1	85.6	71.1
5''''	62.8	66.9	62.7	62.7	66.8	62.8	67.0

^a)^ 75 MHz, CD_3_OD; ^b)^ 125 MHz, pyridine-d_5; _^c) ^75 MHz, pyridine-d_5._

## Experimental

### General

Optical rotations were measured with a JASCO DIP-370 digital polarimeter. ^1^H- and ^13^C-NMR spectra were recorded on Varian Unity plus 500 and Varian Gemini 300 spectrometers. Coupling constants (*J*) are expressed in Hz, and chemical shifts are given on a *δ* (ppm) scale with tetramethylsilane as an internal standard. MS were recorded on a JEOL JMS DX-303 spectrometer, and glycerol was used as a matrix for FAB-MS measurement. HR-ESI-MS was preformed on a Q-TOF mass spectrometer (Bruker Daltonics, MA, USA). Column chromatographies were performed with Kieselgel 60 (70 - 230 mesh, Merck), MCI-gel CHP 20P (75 - 150 mm, Mitsubishi Chemical Co. Ltd., Japan), Chromatorex ODS (100 - 200 mesh, Fuji Silysia Chemical). Preparative HPLC (Shimadzu ODS) was performed on a Tosoh apparatus equipped with a CCPM solvent delivery system. Thin layer chromatography (TLC) was performed on precoated Kieselgel 60 F_254_ plates (0.2 mm thick, Merck), and spots were detected by ultraviolet (UV) illumination and by spraying 10% sulfuric acid reagent.

### Extraction and Isolation

Air-dried flowers of *Panax notoginseng* (800 g) purchased from a herbs market in Kunming, Yunnan Province, P.R. China in August, 1997 were extracted three times with methanol at room temperature. After removal of the solvent by evaporation *in vacuo*, the extract was suspended in water and extracted successively with Et_2_O, EtOAc, *n*-BuOH. The H_2_O layer (40.8 g of 168.5 g) was subjected to column chromatography over MCI-gel CHP 20P eluted from 0 % to 100 % MeOH to afford four fractions. Fraction-2 (3.15 g) eluted by (50-60 % MeOH) was chromatographed on MCI-gel CHP 20P (eluted with 50 % to 80 % MeOH) and Chromatorex ODS (eluted from and 50 to 100 % MeOH) to afford a saponin fraction (0.89 g). This fraction were purified by preparative HPLC to give compounds **6** (37.7 mg), **2** (25.3 mg), **9** (32.6 mg), **5** (28.4 mg), **3** (28.4 mg), **4** (11.4 mg), **1** (15.6 mg), **8** (28.6 mg) and **7** (58.4 mg).

*Floranotoginsenoside A* (**1**): C_53_H_90_O_23_, white powder, [α]^D^_25_: -4.93^º^ (MeOH). Negative FAB-MS (*m/z*):1,093 [M-H]^-^; Negative HR-ESI-MS (*m/z*): 1,093.5790 [M-H]^- ^(calculated for C_53_H_89_O_23_, 1,093.5795); ^1^H-NMR (CD_3_OD, 300 MHz): *δ* 0.86, 0.91, 0.93, 1.02, 1.07, 1.33, (3H each, all s, H-19, 30, 18, 21, 29, 28), 1.29 (6H, s, H-26, 27), 4.60 (1H, d, *J* = 8 Hz, H-1'), 4.66 (1H, d, *J* = 8 Hz, H-1'''), 4.82 (1H, d, *J* = 8 Hz, H-1''), 4.94 (1H, s, H-1 '''') 5.71 (2H, m, H-23, 24); ^13^C-NMR data are listed in Table 1. 

*Floranotoginsenoside B* (**2**): C_53_H_90_O_24_, white powder, [α]^D^_25_: -9.50^º ^ (MeOH); Negative FAB-MS (*m/z*):1,091[M-H-H_2_O]^-^; Negative HR-ESI-MS (*m/z*) 1,109.5748 [M-H]^-^ (calculated. for C_53_H_89_O_24_, 1,109.5744); ^1^H-NMR (pyridine-d_5_, 300 MHz): *δ*0.81, 0.94, 0.96, 1.11, 1.28, 1.64, 1.96 ( 3H each, all s, H-19, 30, 18, 29, 28,21,27), 3.27 (1H, dd, *J* = 3, 11Hz, H-3 ), 4.74 (1H, dd-like, H-24 ), 4.92 ( 1H, d, *J* = 8 Hz, H-1' ), 5.00 ( 1H, d, *J* = 8 Hz, H-1'''' ), 5.05 ( 1H, d, *J* = 8 Hz, H-1''' ), 5.38 ( 1H, d, *J* = 8 Hz, H-1'' ); ^13^C-NMR data are listed in Table 1. 

*Floranotoginsenoside C* (**3**): C_53_H_90_O_24_, white powder, [α]^D^_25_: -14.68^º ^(MeOH); Negative FAB-MS (*m/z*):1,091[M-H-H_2_O]^-^; Negative HR-ESI-MS (*m/z*) 1,109.5747 [M-H]^-^ (calculated. for C_53_H_89_O_24_, 1,109.5744); ^1^H-NMR (pyridine-d_5_, 300 MHz): *δ* 0.80, 0.94, 0.94, 1.11, 1.28, 1.62, 1.97,(3H each, all s, H-19, 30, 18, 29, 28, 21, 27), 3.27 (1H, dd, *J* = 3, 11 Hz, H-3), 4.79 (1H, dd-like, H-24), 4.91 (1H, d, *J* = 8 Hz, H-1' ), 5.10 (1H, d-like, H-1'''), 5.39 (1H, d, *J* = 8 Hz, H-1"), 5.66 (1H, s, H-1''''); ^13^C-NMR data are listed in Table 1.

*Floranotoginsenoside D* (**4**): C_53_H_90_O_23_, white powder, [α]^D^_25_: -14.04^º ^(MeOH); Negative FAB-MS (*m/z*):1,093[M-H]^-^; Negative HR-ESI-MS (*m/z*) 1,093.5792 [M-H]^-^ (calculated. for C_53_H_89_O_23_, 1,093.5795); ^1^H-NMR (pyridine-d_5_, 300 MHz): *δ*0.80, 0.93, 0.99, 1.10,1.28, 1.64, 1.95, (3H each, all s, H-19, 30, 29, 28, 21, 27), 3,25 (1H, dd, *J* = 3, 11 Hz, H-3), 4.90 (1H, d, *J* = 8 Hz, H-1'), 5.10 ( Overlapping, H-26 ), 5.09 (Overlapping, H-1'''), 5.40 (1H, d, *J* = 7.4 Hz, H-1"), 5.66 ( 1H, s, H-1''''); ^13^C-NMR data are listed in Table 1.

*Reduction of* **2** *and* **3**: Triphenylphosphine (10 mg) was added to a solution of **2** (10 mg) in MeOH (5 ml). After stirring at room temperature for 4 hr, the solution was evaporated to dryness under reduced pressure, then subjected to silica gel chromatography (CHCl_3_-MeOH-Water: 8:2:0.2) to afford **9** (6.1 mg) whose ^1^H- and ^13^C-NMR spectral data are completely identical with those of gypenoside LXXI. Reduction of **3** (10 mg) in a manner similar to that described for **2** yielded floranotoginsenoside D (**4**, 5.6 mg).

## Conclusions

Among four new darmmarane-type triterpene saponins isolated from notoginseng flowers, compounds **2** and **3** have a hydroperoxyl group. Although hydroperoxydammarane triterpene saponins have been previously isolated from the roots of *Panax notoginseng* [12], this was the first report of the isolation of this kind of saponins from the notoginseng flowers. These results may contribute to better understanding on the chemical characteristics of this medicinal herb. 
